# Differential characterization of stress sensitivity and its main control mechanism in deep pore-fracture clastic reservoirs

**DOI:** 10.1038/s41598-021-86444-3

**Published:** 2021-04-01

**Authors:** Denglin Han, Huachao Wang, Chenchen Wang, Wenfang Yuan, Juan Zhang, Wei Lin, Rongrong Hu

**Affiliations:** 1 School of Geosciences, Yangtze University, Wuhan, 430100 China; 2 Laboratory of Reservoir Microstructure Evolution and Digital Characterization, Yangtze University, Wuhan, 430100 China; 3 Cooperative Innovation Center of Unconventional Oil and Gas, Yangtze University, Wuhan, 430100 China; 4 PetroChina Tarim Oilfield Company, Korla, 841000 China; 5 School of Petroleum Engineering, Yangtze University, Wuhan, 430100 China

**Keywords:** Geology, Structural geology

## Abstract

Stress sensitivity in reservoirs is critical during the exploitation of oil and gas fields. As a deep clastic reservoir under strong tectonic compression, the Ahe Formation in the northern tectonic zone of the Kuqa depression exhibited strong stress sensitivity effect. However, the conventional evaluation method by using permeability damage rate as a constraint restricts the mechanistic understanding of the strong stress sensitivity effect. In this study, morphology of stress sensitivity test curve, coupled with rate change of permeability and extent of irreversible damage in actual sample measurement through micro-CT in-situ scanning, is used to characterize differentially. The strong stress sensitivity effects of the studied intervals can be divided into three types: (1) rapid change in permeability–weak irreversible damage, (2) moderate change in permeability–strong irreversible damage and (3) moderate change in permeability–moderate irreversible damage. The strong stress sensitivity is caused by the micro-pores and micro-fractures, which are widely developed in the studied reservoir. The mechanisms caused by the two types of pore are different. The stress sensitivity effects in micro-fracture-rich reservoirs are characterized by rapid change in permeability and weak irreversible damage. Meanwhile, the stress sensitivity effects in micro-pore-rich reservoirs are manifested as moderate change in permeability and strong irreversible damage. The study shows that the differences in the content of micro-pores and micro-fractures and their reverse mechanisms of stress sensitivity co-create different types of stress sensitivity within the samples. Accordingly, the differences of the stress sensitivity type in macroscopic samples are caused by the competition between the microscopic differences of pore types.

## Introduction

Reservoir sensitivity is proved to be one of the most important restricting factors in oil and gas exploration and exploitation^1–5^. As the reservoir exploitation enters the substantial stage, stress sensitivity is always involved during pressure relief exploitation and fracturing transformation in any wells. Therefore, reservoir damage caused by stress sensitivity draws increasing attention from industry and academia, and has also been considered to be a required process for reservoir evaluation during reservoir exploitation^4,6–9^.

As a strategic replacement area for exploration in the Tarim Basin, the Jurassic Ahe Formation in the northern Kuqa depression, buried deeper than 4500 m, has become a typical representative of deep hydrocarbon reservoirs in China^10–12^. Previous studies on stress sensitivity mainly follow industry standards. However, according to the current evaluation criteria, the stress sensitivity of the Ahe Formation reservoirs is moderate strong to strong. Hence such indistinctive evaluation results cannot be used to reveal the difference in stress sensitivity characteristics of different blocks (intervals). So the sensitization mechanism of strong stress sensitivity effect is not clear. As a result, the differential characterization of strong stress sensitivity effects is an important issue worthy of further investigation.

Stress sensitivity refers to the phenomenon that the permeability of the reservoir changes with the net overlying stress of the rock because of pores or throats deformation, and fractures closure or opening. Hence, pore-throat structure in reservoir has always been an intrinsic controlling factor of the stress sensitivity effect, and it is undoubtedly the best breakthrough for stress sensitivity mechanism analysis^13–18^.

The study here focuses on the permeability change rate characterization based on a series of stress sensitivity testing curves. With the help of micro-CT scan and micro pore-throat analysis, the control mechanism of reservoir pore-throat types with strong stress sensitivity effect is discussed.

## Overview of the study area

**Figure 1:**
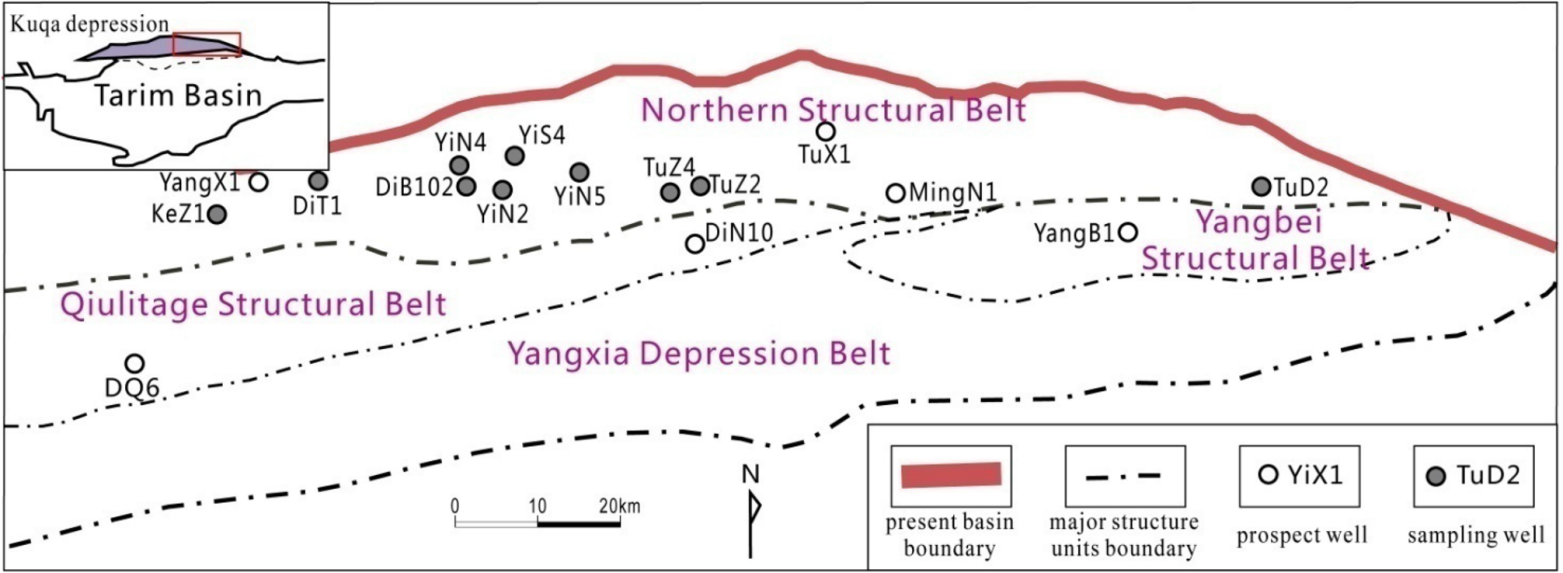
Structural outline map of the north tectonic belt of Kuqa depression and the distribution of the sampling wells.

The Kuqa depression is located in the northern part of the Tarim Basin (Fig. 1), and the main exploration stratum of the northern tectonic belt is the Lower Jurassic Ahe Formation^10,12^. According to the difference of lithology, the Ahe Formation is divided into three lithologic intervals from bottom to top, i.e., the lower sandstone section (J_1_*a*^3^), the middle sandy conglomerate section (J_1_*a*^2^) and the upper sandy conglomerate–mudstone section (J_1_*a*^1^). The lower sandstone section, which is deposited in an underwater distributary channel, is the most important interval because of its high permeability, good sorting and weak heterogeneity. It mainly consists of a suite of grayish white, light grey medium-fine sandstone or siltstone with thick sand body, less mudstone interlayer, and stable regional distribution.

## Analytical methods

In order to make the testing samples as diverse as possible in mineral composition and pore-throat structure characteristics, 13 representative samples from the Ahe Formation were collected from 10 wells in the northern structural belt, the Kuqa depression (Fig. 1). To avoid the influence of porosity and permeability diversity on flow test in reservoir, medium-coarse sandstones are mainly sampled from cores without visible fractures.

### Sample preparation

**Figure 2:**
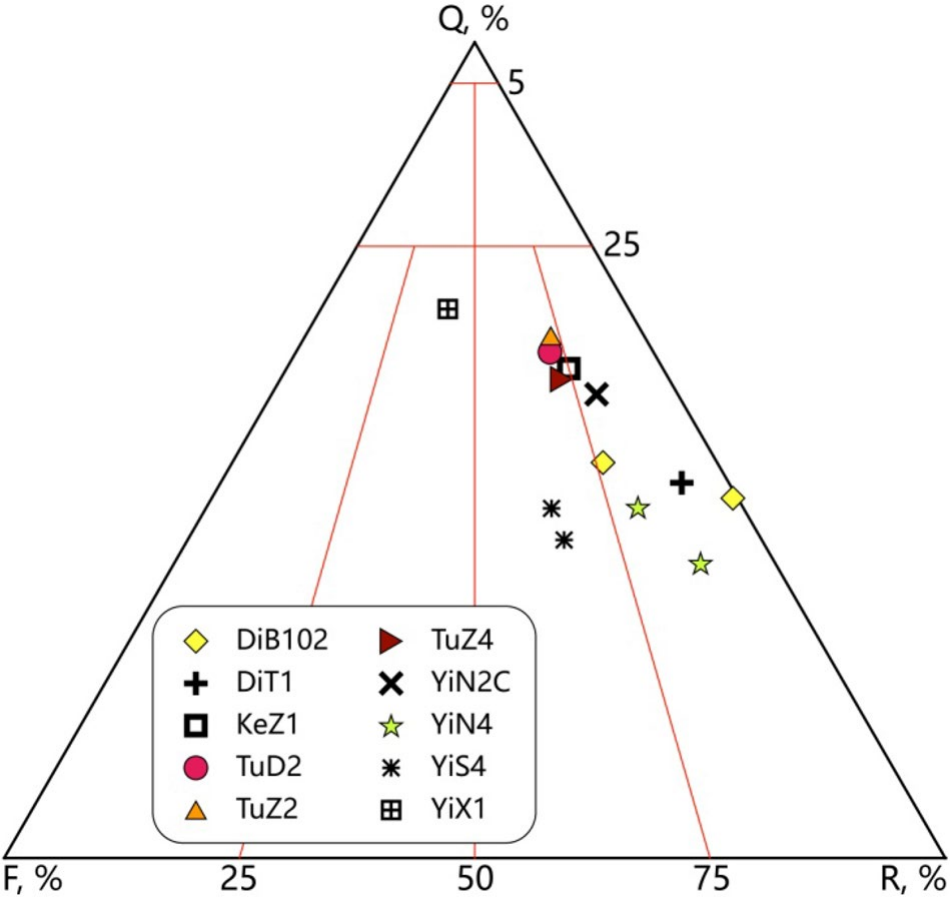
Mineral composition of the rock samples based on microscopic statistics.

During the sampling process, regular rock plug was obtained by drilling along the direction of core diameter with a drill diameter of 2.54 cm. Based on microscopic statistics of the rock samples, the quartz content distributes between 36.0 and 67.3%, which is shown in the mineral composition triangular chart (Fig. 2), the rock component type is lithic sandstones and feldspathic litharenite. For the in-situ stress sensitivity test, the effect of rock component content is relatively small.

### Reservoir stress sensitivity evaluation test

For each sample, a regular rock plug with a length of 4.0 cm and diameter of 2.54 cm was cut from the middle of plug for the reservoir stress sensitivity evaluation test. The real-time permeability parameters under different confining pressures were acquired and the curves of permeability change with pressure were plotted.

### Micro-CT in-situ scanning with confining pressure

The plug (with a diameter of 8 mm and length of 20 mm) of representative samples were drilled from the remaining samples after cutting. Afterwards, continuously increasing confining pressure was applied to these samples by using a specialized holder. At the confining pressure of 2 MPa, 5 MPa, and 15 MPa, corresponding to the initial, intermediate and final stages of the stress-sensitive effect respectively, micro-CT in-situ scanning was separately conducted to the samples to reconstruct their pore-throat structures. The study focuses on the characteristics of different formation pore types in deep clastic reservoir under outer pressure. Due to the pressure increasing limitation and high effective stress in deep reservoir, the effective stress effect is not considered during micro-CT in-situ scanning. And “effective pressure” is taken into consideration as the sensitivity parameter.

**Figure 3:**
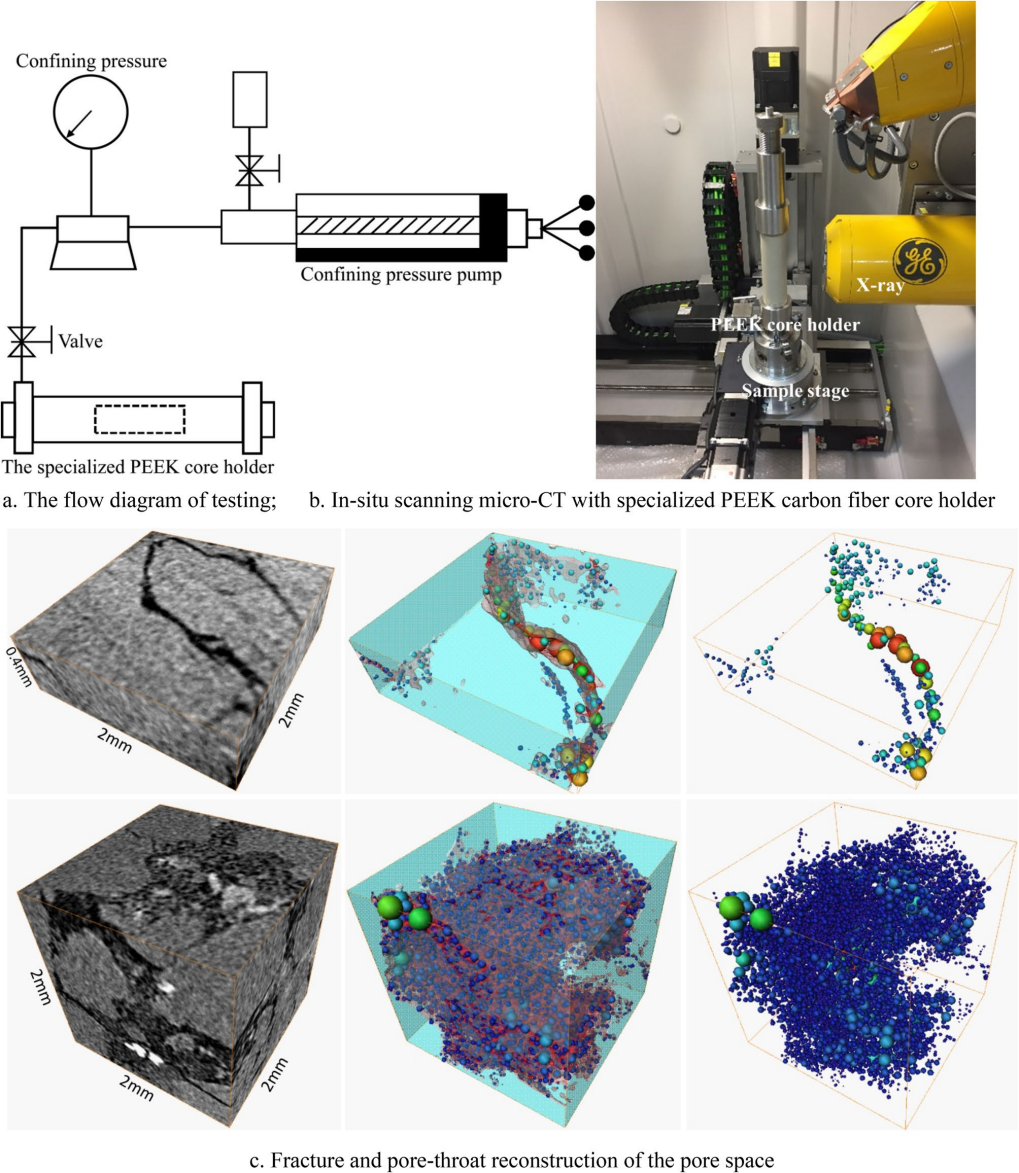
Simulation process of pore-throat reconstruction of the pore space in the studied interval by maximal ball method and micro-CT scan.

The in situ X-ray CT experiments are performed with a micro/nanometer X-ray CT system phoenix v|tome|x s with 0.5 micron of maximum imaging resolution, provided by GE Corporation. The micro-pores we focused in this paper are those with diameter between 2 μm and 30 μm, which could be explored to study the stress sensitivity characterization by micro-CT with maximum imaging resolution 0.5 micron^19–21^. The confining pressure was applied to these sample with a specialized PEEK carbon fiber holder, which could be penetrated by X-ray. The following is the specific operation procedures (Fig. 3).

1. The confining pressure at 2 MPa is added on the specialized PEEK core holder through the confining pressure pump.
2. The valve is turned off and the core holder is taken out separately to fix on the CT sample stage.
3. X-ray is turned on to scan the sample in the PEEK core holder with the rotation of the sample stage.
4. The core holder is taken out from the sample stage after CT scanning with the confining pressure at a certain value and is connected the confining pressure pump.
5. Repeat (1) to (4) for the confining pressure 5 MPa and 15 MPa respectively.

During this process, the maximal ball method was used to determine the aperture of fractures and radius of micro pores^22^. In the maximal ball algorithm, maximal balls are called for the largest inscribed spheres is produced to touch the grain or the boundary while the included spheres are considered as inclusions and removed, which is used to describe the pore space with parameterized topology and geometries. Based on percolation theory in pore network modeling^22^, flow calculations have been performed on the pore network, and flow processes are simulated according to invasion-percolation principles. The parameters to be calculated from pore network flow simulation include absolute permeability and relative permeability, while absolute permeability could be calculated from Darcy equation with single phase flow simulation^22–27^ (Fig. 3).

### Thin section observation

The rest of the remaining plugs was excised and observed by means of cast thin sections.

## Stress sensitivity test and reservoir evaluation

In general, as confining pressure increases, the permeability of the test samples decreases gradually. After reaching critical stress, the change in the permeability of the sample is slowed down, and the rate of change between the real-time permeability and the initial permeability at this time (%) is called the damage rate of stress sensitivity. When the confining pressure gradually decreases, the permeability of the test sample will rise to a certain extent. When the confining pressure is reduced to the initial value (generally 2 MPa), the rate of change (%) between the permeability and the initial permeability at this time is called the irreversible damage rate of stress sensitivity^5,13^.

The formula follows the current China Industrial Standards “Evaluation method of formation sensitivity flow test” (No. SY/T 5358-2010): 

$$K_{n} = \frac{{\mu \cdot L \cdot Q}}{{\Delta p \cdot A}} \times 10^{2}$$

*K*_*n*_: Real-time permeability during pressure increasing process, mD, *μ*: fluid viscosity, mPa·S, *L*: length of the sample, cm, *Q*: volume of fluid passing through the sample per unit time, cm^2^/s, $\Delta p$: difference of pressure between two ends of the sample, MPa, *A*: cross-sectional area of the sample, cm^2^.

$$D_{stn}=\frac{{K_{i} - K_{n} }}{{K_{i} }} \times 100{\text{\% }}$$

$$D_{st} \, = \,max(D_{st1} ,D_{st2} , \ldots ,D_{stn} ).$$

*D*_*stn*_: Permeability change rate under different confining pressure, dimensionless, *K*_*i*_: Permeability under initial pressure without confining pressure (2 MPa), mD, *D*_*st*_: Damage rate of stress sensitivity, dimensionless.

$$D_{ir}=\frac{{{\text{K}}_{i} - K_{i}^{,} }}{{{\text{K}}_{i} }} \times 100{\text{\% }}$$

D_*ir*_: Irreversible damage rate of stress sensitivity, dimensionless, ${K}_{i}^{,}$: Permeability under initial pressure after the confining pressure decreasing process, mD.

**Figure 4:**
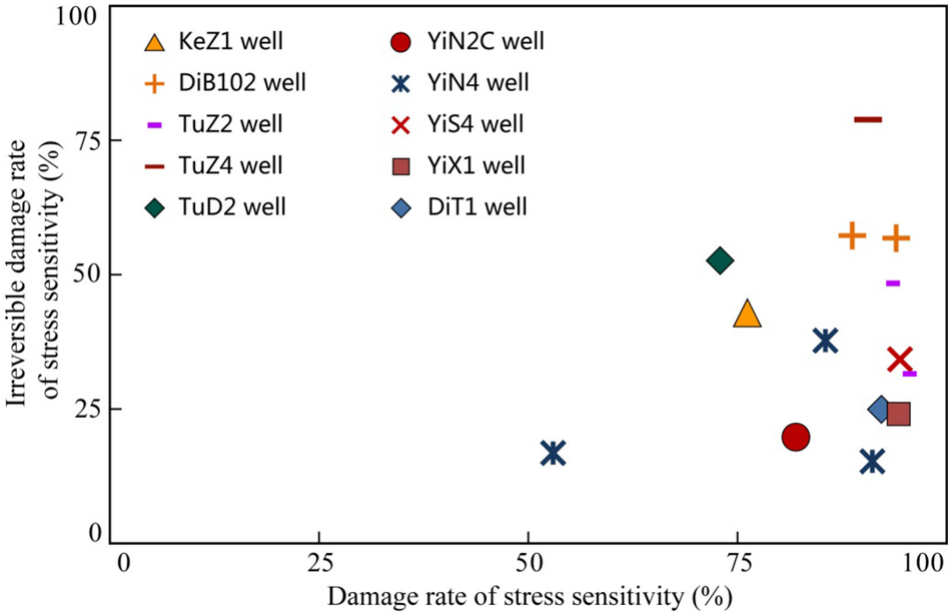
Correlation between stress sensitivity damage rate and irreversible damage rate of the samples in the studied samples.

The damage rates of stress sensitivity measured by different samples ranged from 53.0 to 94.7% with an average of 85.3% (Table 1). On the whole, the stress sensitivity effects of the study interval are moderate strong-strong. The measured irreversible damage rate of stress sensitivity ranges from 15.2% to 79.2% with an average value of 37.4%. The irreversible damage rate of each sample varies greatly from one and another, and there is no significant correlation between the damage rate of stress sensitivity and the irreversible damage rate (Fig. 4).

**Table 1:** Datasheet of stress sensitivity test of the samples in the studied interval.

Well name	Depth of sample (m)	Formation of sample	Porosity (%)	Gas log permeability (mD)	Critical pressure (Mpa)	Damage rate of stress sensitivity (%)	Irreversible damage rate (%)	Rate of change 1	Rate of change 2
DiT1	2259.81	J_1_*a*^3^	10.49	0.439	6.60	92.13	25.07	0.344	0.053
YiX1	410.39	J_1_*a*^3^	5.42	0.034	6.20	94.20	24.20	0.32	0.091
KeZ1	4385.2	J_1_*a*^3^	2.32	0.047	13.60	76.10	43.10	0.154	0.03
YiS4	4046.1	J_1_*a*^1^	7.01	1.365	9.46	94.50	34.20	0.215	0.061
YiN4	4607.5	J_1_*a*^1^	3.86	0.323	8.79	53.00	16.70	0.203	0.045
YiN4	4597.3	J_1_*a*^1^	6.85	0.182	8.20	91.19	15.15	0.224	0.063
YiS 4	4006.2	J_1_*a*^2^	7.90	0.170	8.10	85.57	37.69	0.18	0.045
YiN 2C	4758.72	J_1_*a*^1^	6.15	0.023	19.88	81.90	20.00	0.079	0.05
DiB 102	5095.06	J_1_*a*^2^	9.73	1.083	5.40	88.79	57.29	0.247	0.078
DiB 102	5145.54	J_1_*a*^3^	6.48	1.188	10.7	94.04	56.80	0.183	0.103
TuZ 2	4348.2	J_1_*a*^3^	7.66	0.867	9.60	92.71	48.60	0.196	0.077
TuZ 4	4206.7	J_1_*a*^2^	8.36	5.868	8.60	90.54	79.16	0.195	0.072
TuD 2	4136.3	J_1_*a*^1^	10.74	2.750	9.10	72.84	52.85	0.13	0.027

## Characterization of the stress sensitivity test curve

Although the measured data show that the stress sensitivity effect of each sample is relatively consistent (all of the stress sensitivity effects are moderate strong-strong), the difference in the test curve of different samples is more obvious through comparing of the shape of stress sensitivity test curve.

### Damage rate of stress sensitivity

**Figure 5:**
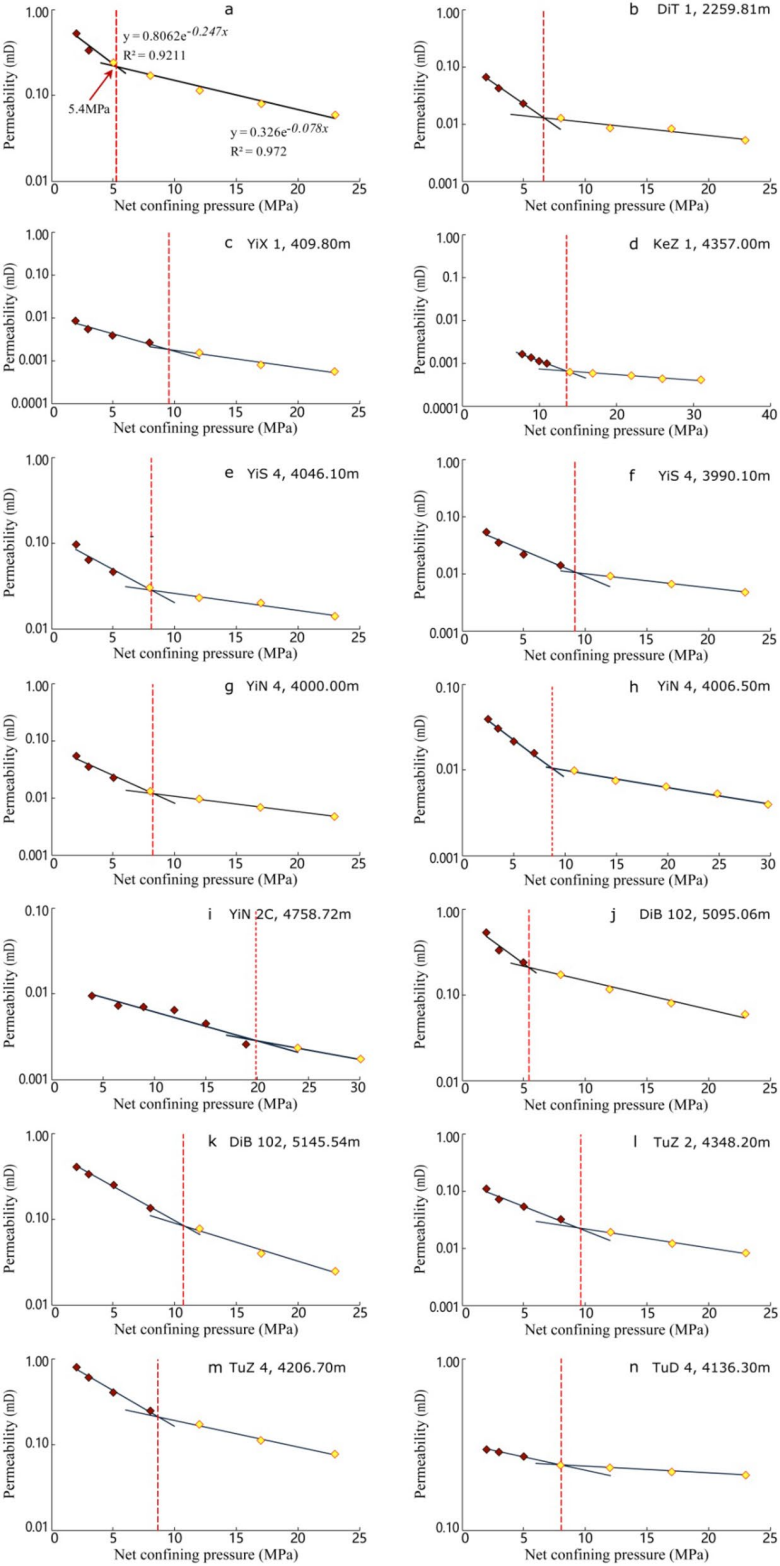
Curve of permeability variation with increasing confining pressure in all studied samples.

Based on the difference in the shape of the curve, the change rate is introduced to characterize the difference in this study. As the confining pressure increases continuously, the difference in the change rate of permeability (the absolute value of the slope of the test curve) is roughly divided into two segments, namely the change rate 1 and the change rate 2 (Table 1, Fig. 5).

The data show that the difference in the change rate 1 segment is the most obvious in the testing curve of all samples. According to the numerical value, it can be roughly divided into three categories, rapid change (change rate ≥ 0.30), moderate change (0.10 ≤ change rate ≤ 0.30) and slow change (change rate ≤ 0.10). Among them, the samples of Well Ditan 1 and Well Yixi 1 show rapid change characteristics, and most of the samples from other single wells show moderate variation characteristics (Table 1).

### Irreversible damage rate

As mentioned above, the damage rate of irreversible stress sensitivity of the samples obtained by the test is significantly different. Therefore, the samples are roughly divided into three types according to the degree of irreversible damage rate in this study, namely, strong irreversible damage rate (≥ 40%),medium irreversible damage rate (25–40%) and weak irreversible damage rate (≤ 25%). Samples from Well Dibei 102, Well Tuzi 2, Well Tuzi 4 and Well Tudong 2 show a strong degree of irreversible damage, and samples of Well Ditan 1 show a weak degree of irreversible damage, while samples from the other single wells exhibit moderate irreversible damage.

Through the analysis of the test curve, the stress sensitivity types of the above samples can be roughly classified into three types:(1) Rapid change in stress sensitivity and weak irreversible damage (mainly in Well Ditan 1); (2) Moderate change in stress sensitivity and strong irreversible damage (mainly in wells in Kezi, Dibei, Tuzi, and Tudong);(3) Moderate change in stress sensitivity and moderate irreversible damage (mainly in wells in Yishen and Yinan).

## Controlling factors

**Figure 6:**
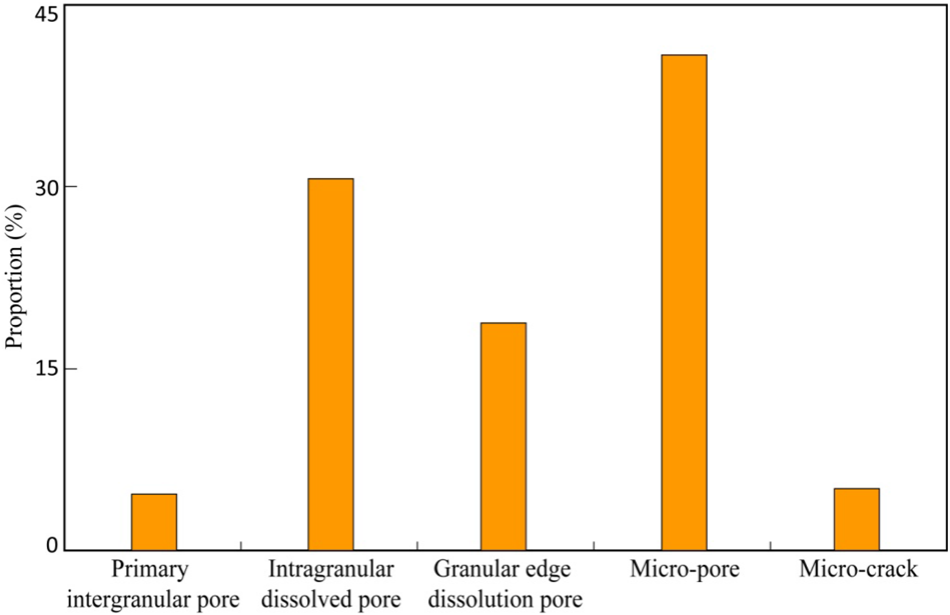
Relative proportion of different pores types in the studied interval (%).

The pore-throat structure of reservoir is the best point cut for the analysis of the difference in stress sensitivity effects. Therefore, pore-throat structure of samples from each well have been compared this study. The pore types of the Jurassic Ahe Formation in the Kuqa depression are diverse, mainly including micro-pores, intra-granular dissolved pores, and grain-margin dissolved pores, which are followed by micro-fractures and primary inter-granular pores^28,29^ (Fig. 6). As the grains support each other, primary inter-granular pores, intra-granular pores, and grain-margin dissolved pores are not very sensitive to the change of confining pressure. Therefore, these three types of pores have not exerted the controlling impact on the difference in stress sensitivity.

**Figure 7:**
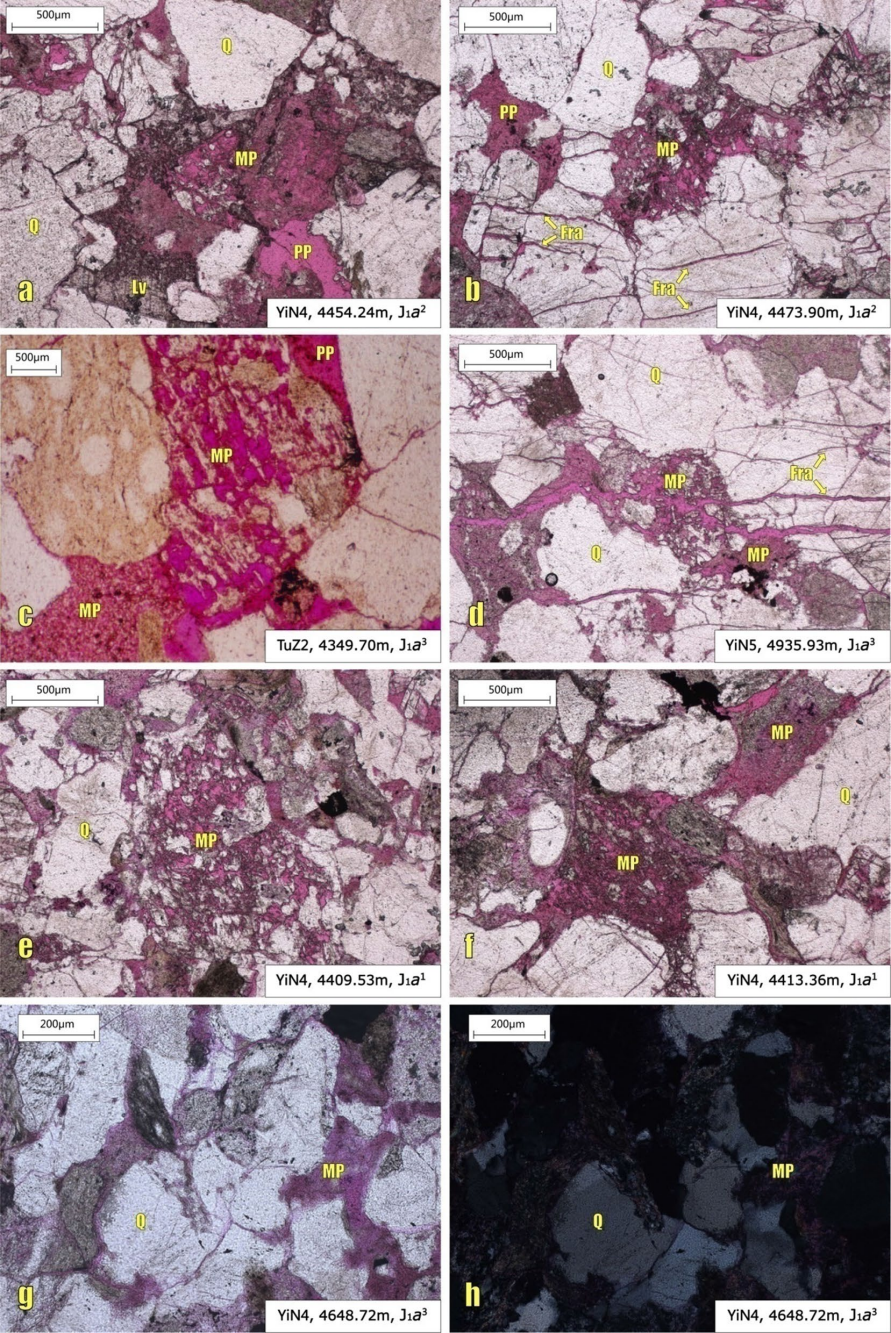
Microscopic plates of different pore types in the studied interval. Fra: Micro-fracture; Lv: Volcanic debris; MP: micro-pore; PP: primary inter-granular pores; Q: quartz.

**Figure 8:**
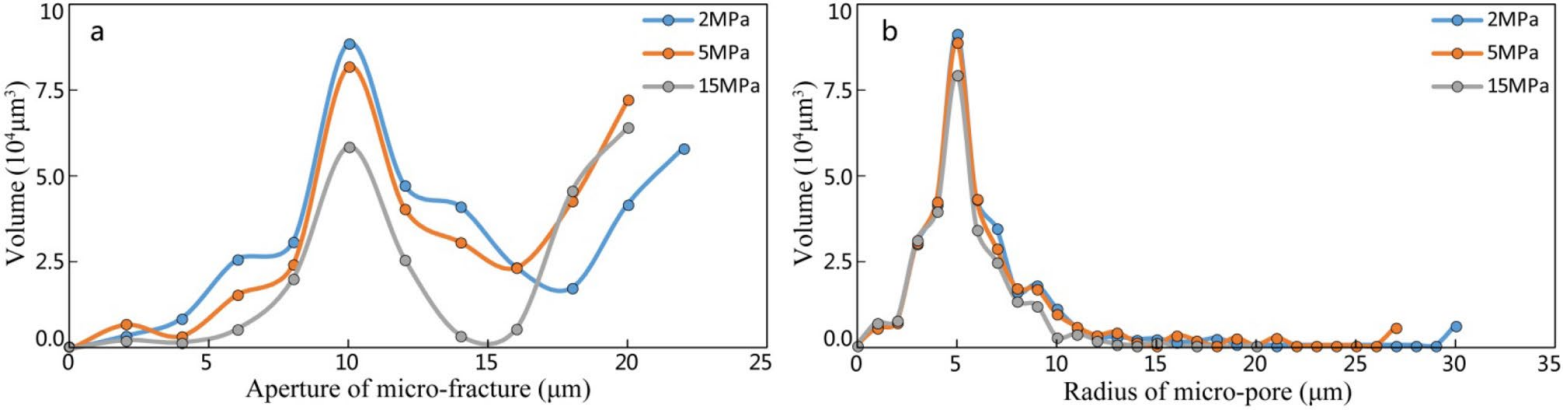
Volume change of different pores with increasing confining pressure in the studied interval.

In the studied interval, micro-pores are widely developed (Figs. 7, 8), and two main causes account for their formation. First, dense micro-pores can be formed by strong erosion of grains (mainly metamorphite lithic fragments)^29,30^ (Fig. 7a–f).Second, fine inter-crystalline pores can be formed by the dissolution of inter-granular interstitial materials (mainly matrix)^28^ (Fig. 7g,h). As a sandstone reservoir under strong tectonic compression, both macro-fractures which can be observed in the cores and micro-fractures at the microscopic level are well developed^12,28,31^ (Fig. 7b,d).

The stress-sensitive effects of micro-fractures and micro-pores with confining pressure changes will be described here.

### Process of increasing confining pressure

**Figure 9:**
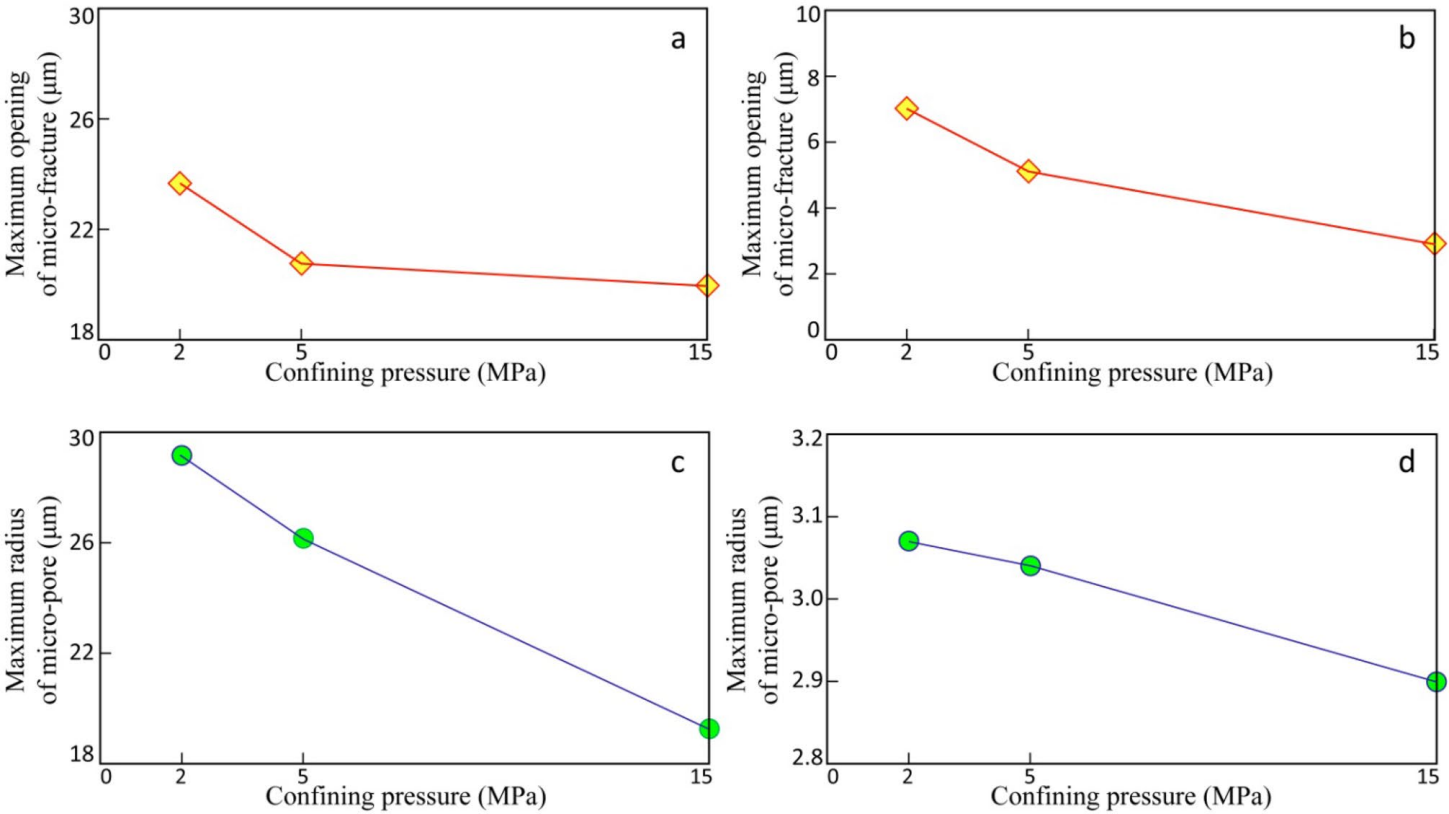
Variation of the aperture of micro-fractures and the radius of micro-pores with increasing confining pressure in the studied interval by maximal ball method and micro-CT scan.

The results of micro-CT scan and the following construction for pore-throat structure show that during the application of confining pressure to the samples (from 2 to 5 MPa), micro-fractures in the samples close rapidly with the sharp decrease of micro-fracture apertures (Fig. 9a,b) and the volume of micro-fractures shrinks obviously (Fig. 9a). As the confining pressure increases to 15 MPa, the closure rate of micro-fractures gradually slows down, while the decrease of micro-fracture aperture is not obvious, and the micro-fracture volume does not change significantly. From macroscopic view, with the increase of confining pressure, the permeability of the samples also shows a clear trend from the initial sharp change to the late slow change.

The reducing of micro-fractures is due to the factor, that the development of micro-pores destroys the rigid structure of the grains or matrix where micro-pores are located, thereby exhibiting plastic properties to a certain extent. During the increasing of confining pressure (from 2 to 5 MPa), micro-pores in the grains or matrix are squeezed and the pore radius reduces continuously (Fig. 9c,d), while the micro-pore volume shrinks (Fig. 9b). As the confining pressure increases continuously, the slow shrinkage of micro-pore volume has not stopped. Macroscopically, as the confining pressure increases, the permeability of the samples decrease at a low speed continuously.

### Process of confining pressure release

When the confining pressure releases, the closed micro-fractures under initial stress will be opened to some extent due to elastic deformation. The previously reduced permeability is also restored to a certain extent, thus showing a relative weak degree of irreversible damage. Meanwhile, the micro-pores that are initially squeezed are difficult to recover due to its plasticity, so that the reduced permeability of the sample is difficult to reverse, showing a relative strong irreversible damage rate.

Both pore types can restrict stress sensitivity effect of the samples significantly, and two kinds of sensitization mechanisms can illustrate their exist significant difference. From the perspective of curve morphology of sensitive test, micro-pores tend to be moderate stress sensitive damage and strong irreversible damage, while micro-fractures tend to be rapid stress sensitive damage and weak irreversible damage. For the test samples, the stress sensitivity effect is the macroscopic result of the combination of the two different mechanisms above.

## Discussion

Although both micro-pores and micro-fractures are common in the samples, the buried depth of the study interval varies greatly, covered the mid-deep layers (2000–4500 m) and deep layers (> 4500 m). The formation of micro-fractures is mainly attributed to compaction of the rigid particles, such as quartz particles^12^. And the micro-pores are mainly produced by the incomplete dissolution of unstable components in the reservoir (Fig. 7)^28,29^.

**Figure 10:**
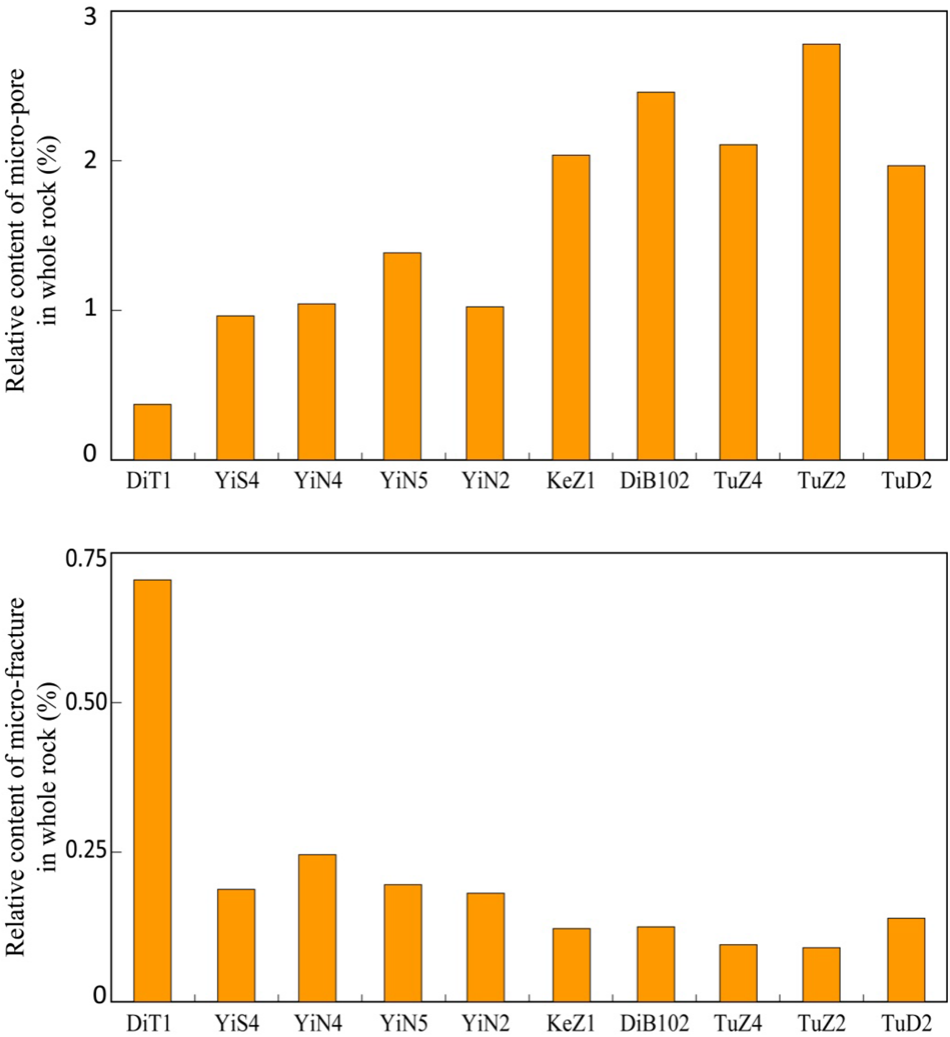
Relative proportion of micro-fractures and micro-pores in the studied interval (%).

Therefore, the types of pore spaces in the study intervals from different wells are significantly different (Fig. 10). According to the difference of micro-fracture and micro-pore content and their different sensitization mechanisms, and the distribution of the study area and single wells, the selected measured samples can be roughly divided into three types:

1. Low micro-pore content and high micro-fracture content characteristics are represented by Well Ditan 1. In the process of stress sensitivity test, with the increase of confining pressure, the rate of permeability reduction is fast. However, with the confining pressure releasing in the later period, the permeability of the samples increase gradually, resulting in low degree of irreversible damage. This type is characterized by rapid stress sensitivity change and weak irreversible damage.
2. Medium micro-pore content and medium micro-fracture content characteristics are represented by wells in the Yinan block and Yishen block. During the stress sensitivity test, the rate of permeability reduction is moderate with the increase of confining pressure. Then as release of confining pressure, the permeability of the samples recovers gradually, resulting in moderate degree of irreversible damage ultimately. This type is characterized by moderate-speed stress sensitivity change and moderate irreversible damage.
3. High micro-pore content and low micro-fracture content characteristics represented by Well Kezi 1, Well Dibei 102, Well Tuzi 1 and Well Tudong 2. During the stress sensitivity test, the permeability decreases moderately with the increase of confining pressure. As the release of confining pressure later, the permeability of the samples recovers gradually, resulting in strong degree of irreversible damage. This type is characterized by moderate-speed stress sensitivity change and strong irreversible damage.

It is important to avoid the stress sensitivity during the occurrence of oil and gas reservoir. In this paper, the mechanisms of stress sensitivity in deep clastic reservoirs are studied from stress sensitivity damages and irreversible damages with different pore space types. The geological significance includes two parts: (a). The formation stress sensitivity type and effect could be predicted from pore types. (b). For deep fractured formation, it shows strong stress sensitivity, however, the irreversible damage is weak. During the occurrence of oil and gas reservoir, it could relax the restriction condition of stress range and replenish the formation pressure with reservoir reconstruction measures, which could compensate the permeability damage caused by stress sensitivity before. Also, reservoir stress may show different sensitivity characteristics for different fluids such as oil and water or gas and water, which could be further studied with more CT scanning sample data in the future.

## Conclusions

1. The study interval shows a strong stress sensitivity effect and different degrees of irreversible damage in general. Based on the quantitative analysis on shape of stress sensitivity testing curve, the stress sensitivity effects in the studied sample suite can be divided into three types according to the difference of stress sensitivity damage rate and irreversible damage degree: (i)rapid stress sensitivity change and weak irreversible damage, (ii)moderate stress sensitivity change and strong irreversible damage, and (iii)moderate stress sensitivity change and moderate irreversible damage.
2. The difference between types of stress sensitivity effect is controlled by the diversity in pore types in reservoirs. Micro-pores and micro-fractures, as two kinds of pore spaces widely developed in the reservoirs, are particularly significant in controlling the effect of stress sensitivity, resulting in opposite stress sensitivity mechanisms. In reservoirs rich in micro-fractures, the stress sensitivity effects are characterized by rapid stress sensitivity damage and weak irreversible damage. In reservoirs rich in the micro-pores, the stress sensitivity effects are characterized by moderate stress sensitivity damage and a strong degree of irreversible damage.
3. The difference in the type of stress sensitivity effect of macroscopic samples is the external manifestation of the competing effect between the two different sensitization mechanisms. The differences in micro-pore content and micro-fracture content in the sample jointly lead to the diversity of stress sensitivity.
